# MiR-155 Has a Protective Role in the Development of Non-Alcoholic Hepatosteatosis in Mice

**DOI:** 10.1371/journal.pone.0072324

**Published:** 2013-08-21

**Authors:** Ashley M. Miller, Derek S. Gilchrist, Jagtar Nijjar, Elisa Araldi, Cristina M. Ramirez, Christopher A. Lavery, Carlos Fernández-Hernando, Iain B. McInnes, Mariola Kurowska-Stolarska

**Affiliations:** 1 Institute of Cardiovascular & Medical Sciences, College of Medical, Veterinary and Life Sciences, University of Glasgow, Glasgow, United Kingdom; 2 Institute of Infection, Immunity and Inflammation, College of Medical, Veterinary and Life Sciences, University of Glasgow, Glasgow, United Kingdom; 3 Marc and Ruti Bell Vascular Biology and Disease Program, New York University School of Medicine, New York, United States of America; University of Tor Vergata, Italy

## Abstract

Hepatic steatosis is a global epidemic that is thought to contribute to the pathogenesis of type 2 diabetes. MicroRNAs (miRs) are regulators that can functionally integrate a range of metabolic and inflammatory pathways in liver. We aimed to investigate the functional role of miR-155 in hepatic steatosis. Male C57BL/6 wild-type (WT) and miR-155^−/−^ mice were fed either normal chow or high fat diet (HFD) for 6 months then lipid levels, metabolic and inflammatory parameters were assessed in livers and serum of the mice. Mice lacking endogenous miR-155 that were fed HFD for 6 months developed increased hepatic steatosis compared to WT controls. This was associated with increased liver weight and serum VLDL/LDL cholesterol and alanine transaminase (ALT) levels, as well as increased hepatic expression of genes involved in glucose regulation (*Pck1, Cebpa*), fatty acid uptake (*Cd36*) and lipid metabolism (*Fasn, Fabp4, Lpl, Abcd2, Pla2g7*). Using miRNA target prediction algorithms and the microarray transcriptomic profile of miR-155^−/−^ livers, we identified and validated that *Nr1h3* (LXRα) as a direct miR-155 target gene that is potentially responsible for the liver phenotype of miR-155^−/−^ mice. Together these data indicate that miR-155 plays a pivotal role regulating lipid metabolism in liver and that its deregulation may lead to hepatic steatosis in patients with diabetes.

## Introduction

Non-alcoholic fatty liver disease (NAFLD) is an increasing health problem in obese individuals in developed countries, and recent studies suggest an association between the presence of NAFLD and diabetes risk [1], [2]. A spectrum of liver tissue pathology exists, comprising hepatic steatosis characterized by the deposition of lipid droplets in hepatocytes, through to non-alcoholic steatohepatitis (NASH) associated with hepatocyte death, inflammation and fibrosis. Advanced disease may progress to cirrhosis and hepatocellular carcinoma (HCC).

The pathogenesis of NAFLD is often rationalized as a ‘double-hit’, whereby diet-induced hepatocellular lipid accumulation presents the ‘first-hit’, followed by a ‘second-hit’ in which pro-inflammatory mediators induce inflammation, hepatocellular injury, and fibrosis [3]. Kupffer cell activation and recruitment of monocytes into damaged liver facilitates pro-inflammatory cytokine release that in turn promotes lipid accumulation, increased inflammation and aberrant fibrosis. The post-transcriptional gene regulatory mechanisms that integrate inflammation and lipid dysregulation in NAFLD are currently poorly understood but could offer significant therapeutic opportunity once elucidated.

MicroRNAs (miRs) are small, non-coding, endogenous RNA molecules (22 nucleotides long) that act as critical post-transcriptional regulators of many biological processes. They function by binding to complementary sequences in the 3′UTRs of specific target mRNAs, usually resulting in gene silencing [4]. Recently, a role for miRNAs in liver disease has been proposed: hepatic expression profiling has revealed temporal changes in miRNA expression in human and murine NAFLD, and identified several differentially expressed miRNAs including miR-21, miR-34a, and miR-122 [5]. In addition, it has been shown that hepatic miR-155 expression was increased in murine models of NASH and HCC, and its expression correlated with disease severity [6], [7]. In line with increased miR-155, the miR-155 target genes CCAAT/enhancer-binding protein beta (*Cebpb)* and suppressor of cytokine signaling 1 *(Socs1)* were decreased. MiR-155 is a multi-functional miRNA known to regulate numerous biological processes including hematopoiesis, inflammation, immunity, atherosclerosis, and cancer (reviewed in 8). However, the functional role of miR-155 in liver homeostasis is unknown. Here we report that absence of miR-155 in mice fed high fat diet was associated with significantly increased hepatic steatosis and serum VLDL/LDL cholesterol levels. miR-155 regulates cholesterol and fatty acid metabolism pathways in liver by directly targeting liver X receptor alpha (LXRα), a transcriptional regulator of many genes in liver lipid metabolism [9]. Thus, our data directly implicate miR-155 in liver homeostasis and its deregulation as a pivotal factor in the pathogenesis of fatty liver disease.

## Experimental Procedures

### Animal Experimentation

Male C57BL/6 wild-type (WT) mice and miR-155^−/−^ mice (Jackson Labs) were bred in-house in a pathogen-free facility and fed normal chow or high fat diet (HFD; 0.15% cholesterol and 21% lard, Special Diet Services) ad libitum from 6 weeks old. Male *ob/ob* mice (Jackson Labs) were fed normal diet from 5 weeks old for 4 weeks. All experiments were approved by the University of Glasgow Animal Procedures and Ethics Committee and performed in strict accordance with UK Home Office guidelines under the Animals Scientific Procedures Act 1986. All efforts were made to minimize animal suffering and the number of animals used was kept to a minimum by the experimental design.

### Insulin Tolerance Tests (ITT)

ITT were carried out 1-day before cull of mice. Briefly, mice were fasted for 4 hrs (ITT), injected intraperitoneally with insulin (0.75 U/kg, Sigma) in 25 mM Hepes. A tail vein blood sample was taken before injection and 30, 60, and 90 minutes after injection for determination of blood glucose using Accu-chek Compact test strips (Roche Diagnostics).

### Morphometric and Immunohistochemical Analysis of Livers

The mice were killed after an overnight fast. Livers were fixed in 10% formalin for 24 hours or kept freshly frozen. Formalin-fixed paraffin-embedded liver sections (5 µm) were stained with hematoxylin and eosin (H&E). Oil red O staining was carried out on frozen liver sections (10 µm). Immunohistochemistry was performed using anti-mouse F4/80 (Serotec) for macrophages, followed by incubation with the ImmPRESS reagent and detection with DAB (Vector Laboratories).

### *In situ Hybridization* on Paraffin-embedded Tissues

After de-paraffinization, sections were treated with proteinase-K (5 µg/mL) for 30 min at 37°C. Hybridization with 5 nmol Locked Nucleic Acid 5′ and 3′ digoxigenin (DIG)-labeled scramble (AGAGCTCCCTTCAATCCAAA) or miR-155–specific (TATCACAATTAGCATTAA) probes (both from Exiqon) was performed at 48°C for 1 h. After hybridization, sections were washed SSC (5x, 1x then 0.2x each for 2 washings of 5 mins) at 48°C and a final wash in 0.2x SSC at RT for 5 min. Next, sections were blocked with DIG Wash and Blocking buffer (Roche) in a humidifying chamber for 15 min at RT. Slides then were incubated with alkaline phosphatase-conjugated sheep anti-DIG antibody (1∶800; Roche) in blocking solution supplemented with 2% sheep serum for 1 h at RT. Sections were incubated nitro-blue tetrazolium (NBT) and 5-bromo-4-chloro-3′-indolyphosphate (BCIP) solution (Roche) for 2 h at 30°C. To stop the reaction, slides were washed twice for 5 min with buffer containing 50 mM Tris-HCl, 150 mM NaCl, and 10 mM KCl. Nuclear counterstaining was performed using Nuclear Fast Red (Vector). Liver tissues were evaluated using a numerical score based on the number of positive cells in the section (three different fields in each section), with a score of 0 indicating no positive cells; 1 indicating <10% positive cells; 2 indicting 10–50% positive cells; and 3 indicating >50% positive cells.

### Liver Lipids

Lipids were extracted from 1 mg of tissue using Isopropanol at 4°C overnight in a rotating glass tube. After extraction the lipids were dried with N_2_ and then resuspended in isopropanol. Protein quantification was carried out using RC DC Protein Assay Kit II (BioRad). Triglyceride and cholesterol content were measured using kits from Wako Diagnostics according to the manufacturer’s protocols.

### Flow Cytometry

Livers were collagenase (C6885, Sigma) digested for 30 minutes at 37°C with shaking and a single cell suspension prepared following filtration through a 100 µm cell strainer. Cells were resuspended in FACS buffer (PBS, 2% FCS, 2 mm EDTA) then incubated with Fc-Block (BD Biosciences) for 15 minutes before staining with F4/80-APC (eBioscience) and CD45-PE (BD Biosciences) or isotype controls. Cells were analyzed on a BD FACS Calibur with CellQuest Pro Software™.

### Serum Analysis

Total serum cholesterol, VLDL/LDL and HDL, and triglyceride levels (mg/dL) were measured by EnzyChrom™ assay kits (Universal Biologicals). Metabolic proteins were analyzed using the mouse adipocyte kit (Insulin, Resistin, Leptin) (Millipore) on the Bio-Plex 100 system (Bio-Rad). Alanine Transaminase (ALT) levels were measured in serum using an ALT Activity Assay Kit (Camridge Bioscience).

### MACS Bead Selections

For macrophage isolation CD11b^+^ cells were selected by labeling with CD11b beads (Miltenyi) and positively selected on an AutoMACs separator. Macrophage populations were confirmed to contain >98% CD45^+^F4/80^+^ cells prior to further experiments.

### Quantitative PCR

Total RNA from frozen liver was prepared by tissue homogenization in Qiazol (Invitrogen) followed by purification with miRNeasy kits (Qiagen). The miScript Reverse Transcription Kit (Qiagen) was used for cDNA preparation. TaqMan Gene Expression Assays (Applied Biosystems) or miScript primer assays (Qiagen) were used for determination of gene expression. The expression of U6B snRNA or TATA binding protein (TBP) was used as endogenous controls and data analyzed on the ABI7900HT machine with SDS 2.2 software. Data is presented as RQ values (2^−ΔΔCT^) with expression relative to endogenous control and WT control sample.

### Microarray Analysis

Total RNA from frozen liver was prepared by homogenization in Qiazol (Invitrogen) followed by purification with miRNeasy kits (Qiagen). RNA quality and quantity was assessed on a Nanodrop (Thermo Scientific) and Agilent 2100 Bioanalyzer. An Affymetrix GeneChip® Mouse Gene 1.0 ST Array was performed using total RNA from three independent biological replicates. The microarray data produced in this study is MIAME compliant (http://www.mged.org/Workgroups/MIAME/miame.html) and it has been submitted to the ArrayExpress database (www.ebi.ac.uk/arrayexpress) under accession No E-MEXP-3932. CEL files were analyzed using R and Bioconductor [10]. Quality control was carried out using package *arrayQualityMetrics* [*11*] before and after RMA pre-processing using the *Oligo* package [12]. One suspect sample was flagged and removed from further analysis. Differential gene expression analysis was carried out using *oneChannelGUI* [13] and Rank Product statistic [14], which is particularly suited to small data sets. A pfp (predictor of false positive) value of 0.05 was chosen to determine differentially expressed genes. Ingenuity Pathway Analysis (IPA) was used for functional and canonical pathway analysis. The significance of association between differentially expressed genes and canonical pathways was determined using Fisher’s exact test to test the probability that the association between genes and pathway occurred by chance alone. The P-value generated was then corrected with Benjamini-Hochberg correction for multiple testing.

### MiR-155 Target Prediction and Validation

MiR-155 target prediction was carried out using the algorithms TargetScanHuman V6.2 (http://www.targetscan.org/vert_61/), PicTar (http://pictar.mdc-berlin.de) and miRTarBase (http://mirtarbase.mbc.nctu.edu.tw). We then compared predicted targets and the miR-155 target database of IPA (TargetScan, Tarbase targets and other literature based targets) with our transcriptomic data to choose candidate genes for further validation (Table S3). In order to validate the candidate miR-155 predicted targets, luciferase reporter assays were carried out. Briefly, potential miR-155 miRNA recognition elements (MREs) were amplified from relevant genomic DNA using PfuUltra II (HS) (Agilent). Primers (Table S4) were designed incorporating *Pme*I and *Sal*I in the forward and reverse primers respectively. Candidate MREs were cloned into the same sites downstream of the Renilla Luciferase gene in pmiRGLO (Promega). HEK293 cells were co-transfected with 0.2 µg pmiRGLO containing potential miR-155 MREs and 40 nM miR155 mimic or scrambled mimic control, using Attractene (Qiagen). Luciferase activity was measured 24 hrs later using the Dual-GLO Luciferase assay system (Promega). The miR-155 seed region of mouse *Nr1h3* was mutated using QuikChange Lightning kit (Agilent) and the primers:

Fw GAAGGAGAGAGCCTTGCGTAggatccAGGGAGAGTC;

Rev GACTCTCCCTggatccTACGCAAGGCTCTCTCCTTC.

### Macrophage Transfection Experiments

CD11b^+^ cells from WT and miR-155^−/−^ livers were transfected with miR-155 or control mimics (25 nM; Dharmacon: mmu-miR-155: UUAAUGCUAAUUGUGAUAGGGGU; control mimic cel-67: UCACAACCUCCUAGAAAGAGUAGA) or anti-mmu-miR-155 (MIN0000165) or control anti-miR (1027271, both 25 nM, Qiagen) with N-Ter transfection reagent (Sigma). After 24 hours whole lysate was prepared using M-PER (Thermo Scientific) lysis buffer. Total protein (10 µg) was ran on 10% SDS Page gel followed by transfer to PVDF membrane and incubation with rabbit anti-mouse/human LXRα (2 µg/ml; Lifespan Biosciences) or anti-mouse/human β-actin (1 µg/ml, Santa Cruz Biotechnology).

### Statistical Analysis

All data are mean ± standard error of mean (SEM). Statistics were performed using unpaired Student’s *t* tests, Mann-Whitney or ANOVA, with GraphPad Prism Software®.

## Results

### Expression of Liver miR-155 is Increased in Murine Models of Diet-induced Obesity

**Figure 1:**
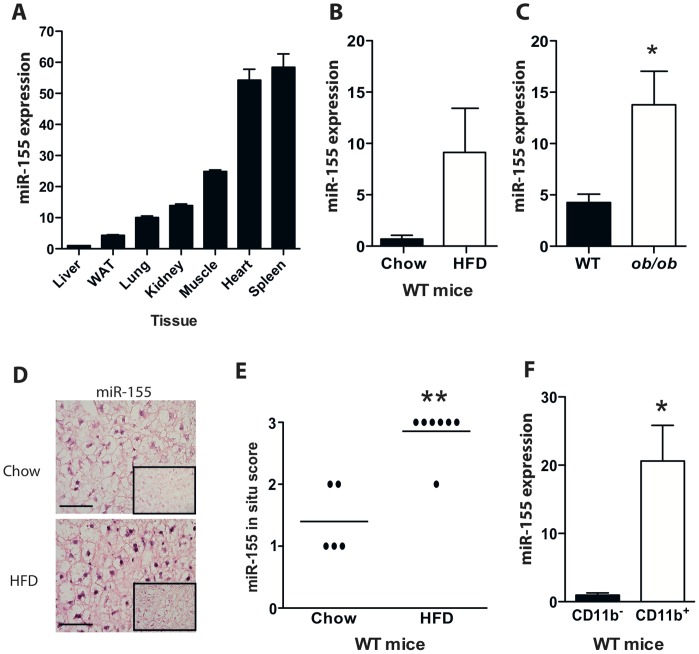
Liver expression of miR-155 is increased in murine models of obesity. Expression of miR-155 by qRT-PCR in: (**A**) Tissues from 8-week old WT mice fed normal chow (n = 3)(WAT: white adipose tissue); (**B**) Livers from WT mice fed either normal chow or HFD for 24 weeks; (**C**) Livers from WT and *ob/ob* mice fed normal chow for 5 weeks. Data are Means ± SEM, n = 3–5 mice/group. (**D**) Representative sections showing localization of miR-155 in livers by *in situ* hybridization (purple stain, 40x magnification, scale bar 5 µm). Sections counterstained with nuclear red. Scrambled oligo probe was used as a negative control (inset). (**E**) Quantification of miR-155 *in situ* staining in livers from WT mice fed either chow or HFD for 24 weeks (n = 5–7/group). ** p<0.01 Mann-Whitney test. (**F**) Expression of miR-155 in bead-sorted CD11b^+^ macrophages and CD11b^−^ cells from livers of WT mice fed HFD for 24 weeks (n = 3). *p<0.05 Student’s unpaired *t*-test.

MiR-155 expression in normal liver is low in comparison to other solid organs in 8-week old WT mice (Figure 1A), but was upregulated under patho-physiological conditions. Quantitative PCR demonstrated significantly higher hepatic expression of miR-155 in WT mice fed HFD (Figure 1B), and in *ob/ob* mice on normal chow versus their respective controls (Figure 1C). Next we mapped the expression pattern of miR-155 in liver by *in situ* hybridization. Elevated miR-155 tissue expression was observed in livers of mice fed HFD, paralleling PCR expression data (Figure 1D and E). These data confirm and extend previous studies showing that expression of miR-155 is increased in livers of animal models of NAFLD [6], [7].

To identify the cell lineage responsible for upregulated miR-155 expression, we purified hepatic CD11b^+^ macrophages. In WT mice fed HFD for 24 weeks, miR-155 expression was higher in CD11b^+^ macrophages compared to the CD11b^−^ fraction, comprising of all other hepatic cell lineages (Figure 1F). These data suggest that homeostatic effects of miR-155 in liver are likely mediated by macrophages/Kupffer cells, and not by hepatocytes.

### miR-155^−/−^ Mice are Susceptible to Hepatic Steatosis

**Figure 2:**
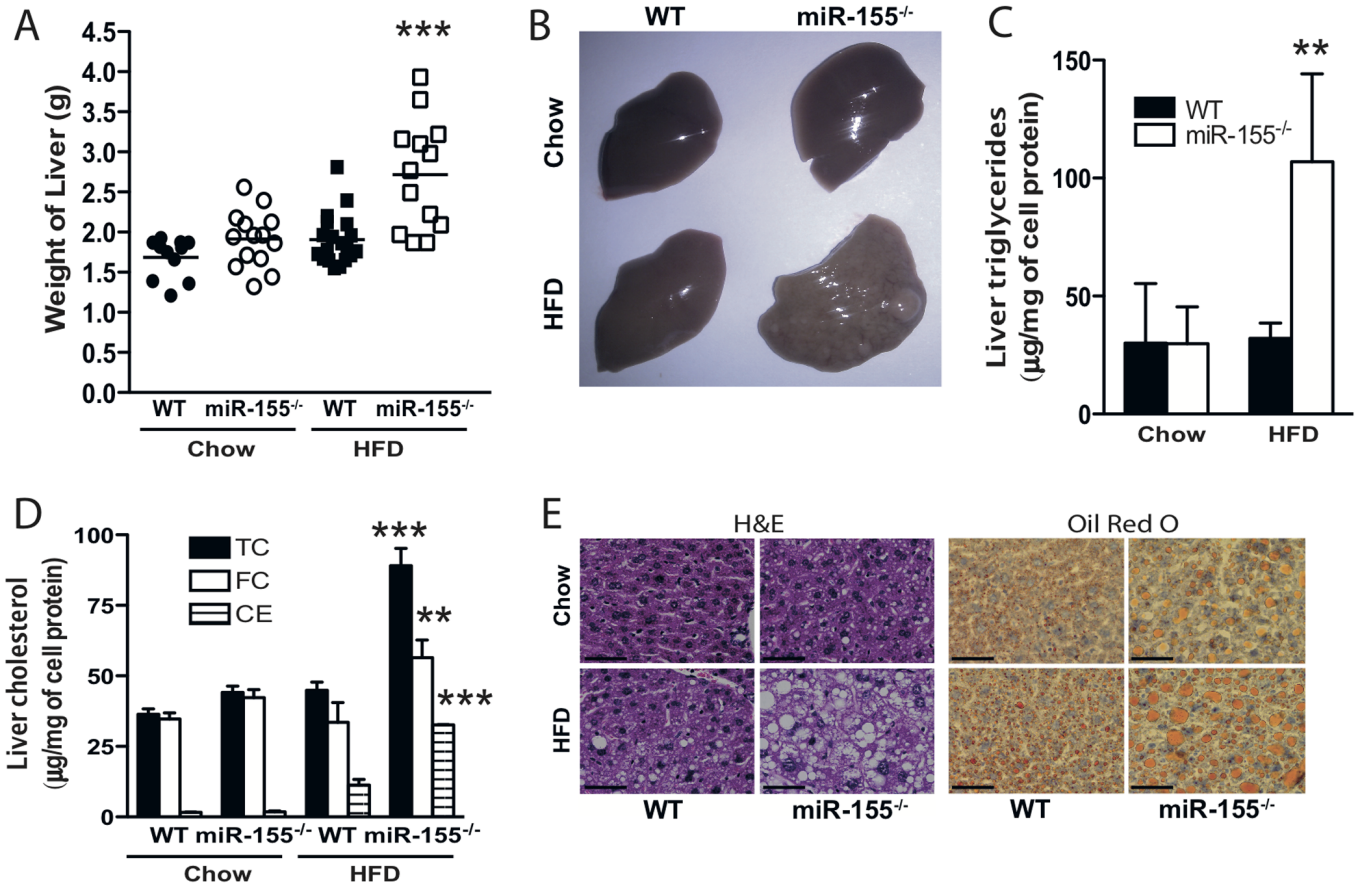
miR-155^−/−^ mice are susceptible to hepatic steatosis. WT or miR-155^−/−^ mice were fed either normal chow or HFD and livers examined at 24 weeks. (**A**) Liver weights (g). (**B**) Gross morphology. (**C**) Total cholesterol (TC), free cholesterol (FC) and cholesterol ester (CE) measurements in liver (µg/mg of cell protein). (**D**) Triglyceride measurements in liver (µg/mg of cell protein). (**E**) Representative H&E, and Oil Red O staining of the livers (40x magnification, scale bar 5 µm). Data are Means ± SEM pooled from 2 independent experiments, n = 13–21 mice/group. ** p<0.01, *** p<0.001 Student’s unpaired *t*-test compared to WT fed HFD.

Since the functional role of miR-155 in development of hepatic steatosis associated with obesity is currently unknown, we next investigated whether miR-155 could integrate inflammatory and lipid signaling in liver *in vivo*, under conditions of diet-induced obesity thereby resulting in hepatic steatosis. We first compared WT and miR-155^−/−^ mice fed chow or HFD for 24 weeks. Deficiency of miR-155 did not alter the final body weight of mice at 24 weeks (Table 1), but mean liver weight was increased by 30% in miR-155^−/−^ mice fed HFD (Figure 2A). The weight of epididymal fat pads, did not differ between groups, indicating a selective effect on liver (Table 1). Changes in the gross appearance of livers from miR-155^−/−^ mice were evident with yellow/brown coloration and visible surface nodules (Figure 2B). Liver triglyceride (Figure 2C) and total cholesterol (TC), free cholesterol (FC) and cholesterol ester (CE) levels were significantly increased in livers of miR-155^−/−^ mice vs WT fed HFD (Figure 2D). Abundant lipid droplets were observed in livers of WT and miR-155^−/−^ mice fed HFD (Figure 2E). Serum levels of the enzyme ALT, indicative of liver damage, were increased in miR-155^−/−^ mice fed HFD (Table 1). Furthermore, serum levels of VLDL/LDL cholesterol were significantly higher in HFD-fed miR-155^−/−^ mice vs WT. Total cholesterol, HDL cholesterol and triglyceride levels in serum did not significantly differ between WT and miR-155^−/−^ mice (Table 1).

**Table 1:** Association of liver weight with murine clinical parameters.

	Chow		HFD	
Parameter	WT	miR-155^−/−^	WT	miR-155^−/−^
Body weight (g)	35.03±0.86	34.48±0.63	39.16±1.36	41.89±1.74
Epididymal fat (g)	0.94±0.04	0.74±0.11	1.88±0.19	1.75±0.20
ALT (U/L)	4.26±0.45	3.42±0.53	5.63±1.29	10.78±1.97*
Total cholesterol (mg/dL)	64.05±4.3	55.43±4.65	93.10±5.84	83.41±8.78
VLDL/LDL cholesterol (mg/dL)	12.92±0.83	13.43±2.95	21.67±1.70	33.20±6.18*
HDL cholesterol (mg/dL)	26.37±5.50	36.31±5.05	61.23±7.01	51.80±9.11
Triglycerides (mg/dL)	52.84±6.99	38.13±19.8	87.01±4.55	118.4±34.4
Insulin (ng/ml)	3.32±0.67	7.01±1.73*	4.98±0.78	10.07±2.55*

Livers were collected from mice at 24 wk of age fed either normal chow or high fat diet (HFD). Alanine transaminase (ALT), cholesterol, triglyceride and insulin values were determined in serum. Data are Means ± SEM pooled from 2 independent experiments: n = 13–21 mice/group.

* p<0.05 Student’s unpaired *t*-test.

Next, to investigate whether glucose and insulin sensitivity were altered in the miR-155^−/−^ mice we measured fasting glucose and insulin, and carried out an insulin tolerance test (ITT) in the mice at week 24. Serum insulin levels were significantly higher in both chow and HFD-fed miR-155^−/−^ mice (Table 1). However, fasting glucose and ITT were similar between WT and miR-155^−/−^ mice (Figure S1).

### Altered Inflammatory and Metabolic Gene Expression in Livers of miR-155^−/−^ Mice

**Figure 3:**
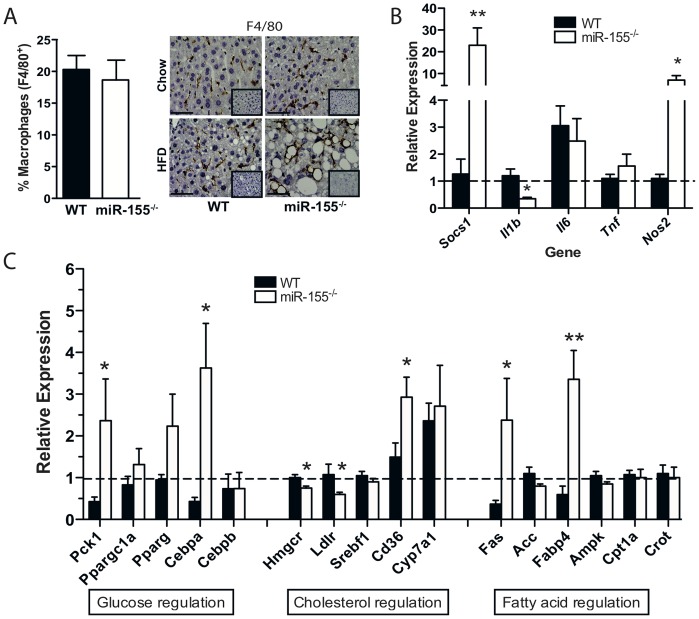
miR-155^−/−^ mice have altered expression of inflammatory and metabolic genes in liver. (**A**) FACS quantification of the % CD45^+^F4/80^+^ macrophages in livers fed HFD and representative F4/80^+^ macrophage staining (brown) and isotype controls (inset) in liver (40x magnification, scale bar 5 µm). qRT-PCR analysis of expression of inflammatory genes (*Socs1, Il1b, Il6, Tnf, and Nos2*) (**B**), and metabolic genes (*Pck1, Ppargc1a, Pparg, Cebpa, Cebpb, Hmgcr, Ldlr, Srebf1, Cd36, Cyp7a1, Fas, Acc, Fabp4, Ampk, Cpt1a, Crot*) (**C**) relative to WT control. Data are Means ± SEM pooled from 2 independent experiments, n = 3–5 mice/group. * p<0.05, ** p<0.01 Student’s unpaired *t*-test compared to WT fed HFD.

To address the molecular mechanisms of enhanced hepatic steatosis in miR-155^−/−^ mice, we examined expression of various inflammatory and metabolic markers in livers and serum of HFD-fed mice. First, we assessed macrophage content of livers using the markers CD45 (pan-leukocytes) and F4/80 (macrophages). Flow cytometric and immunohistochemical analysis demonstrated that CD45^+^F4/80^+^ macrophages did not differ in number between WT and miR-155^−/−^ livers (Figure 3A). Therefore, we assessed the change in expression of liver inflammatory genes. Expression of the miR-155 validated target gene *Socs1* was significantly upregulated in miR-155^−/−^ livers [15] (Figure 3B). We also found significantly increased expression in the genes for inducible nitric oxide synthase (*Nos2*), no change in interleukin-6 (*Il6*) or tumor necrosis factor α (*Tnf*), but reduced expression of interleukin-1β (*Il1b*) in miR-155^−/−^ vs WT mice (Figure 3B).

We then examined the expression of key genes already known to be involved in liver functions. Interestingly, the expression of genes involved in gluconeogenesis (*Pck1, Cebpa*), cholesterol uptake (*Cd36*) and fatty acid synthesis (*Fasn, Fabp4*) were significantly increased in HFD-fed livers of miR-155^−/−^ mice vs WT mice (Figure 3C), indicating that they could be responsible, or contribute to the observed phenotype in miR-155^−/−^ mice. Several other key genes involved in lipid and glucose metabolism were not significantly different (*Ppparc1a, Pparg, Cebpb, Srebf1, Cyp7a1, Acc, Ampk, Cpt1a, Crot*), or were significantly reduced (*Hmgcr*, and *Ldlr*) in miR-155^−/−^ mice on HFD (Figure 3C). In summary, deletion of miR-155 significantly alters the expression of selected inflammatory and metabolic pathways within liver.

### LXRα (Nr1h3): A Lipid Pathway Transcriptional Regulator is a Direct Target of miR-155 in Liver

To gain further insight into the mechanisms underlying such pleiotropic effects of miR-155 in liver we performed a microarray analysis comparing livers from HFD-fed WT and miR-155^−/−^ mice. Using the Rank Products method out of ∼28,853 probesets analyzed we found 573 probeset IDs that were differentially expressed in miR-155^−/−^ vs. WT livers (pfp<0.05). These mapped to 336 genes, of which 286 were upregulated and 50 were downregulated in miR-155^−/−^ livers (Table S1). In order to identify molecular pathways altered within the dataset, the list of differentially regulated genes was uploaded to Ingenuity Pathway Analysis (IPA) software and a Core analysis carried out. Lipid metabolism and cell cycle networks were significantly enriched in livers from miR-155^−/−^ mice, as were networks involved with hepatocellular carcinoma and liver steatosis. Pathway analysis identified the LXR/RXR pathway as the top canonical pathway upregulated in livers from miR-155^−/−^ mice (Table S2).

**Figure 4:**
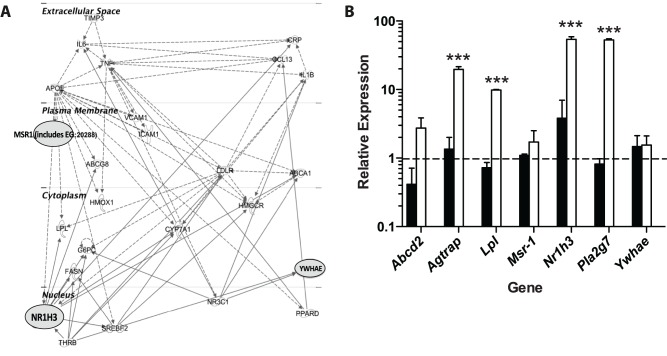
Identification of miR-155 direct targets in liver. (**A**) Interaction map showing liver mRNA that are direct targets of miR-155 by Ingenuity Pathway Analysis that shows genes involved in Lipid Metabolism, Molecular Transport, and Small Molecule Biochemistry. miR-155 direct targets that are up-regulated in miR-155^−/−^ livers are marked in red. (**B**) Expression of the identified target genes *Abcd2, Agtrap, Lpl, Nr1h3* (LXRα) and *Pla2g7* validated by qRT-PCR in WT vs miR-155^−/−^ livers fed HFD (n = 3). Expression is shown relative to WT control. *** p<0.001 Student’s unpaired *t*-test.

**Figure 5:**
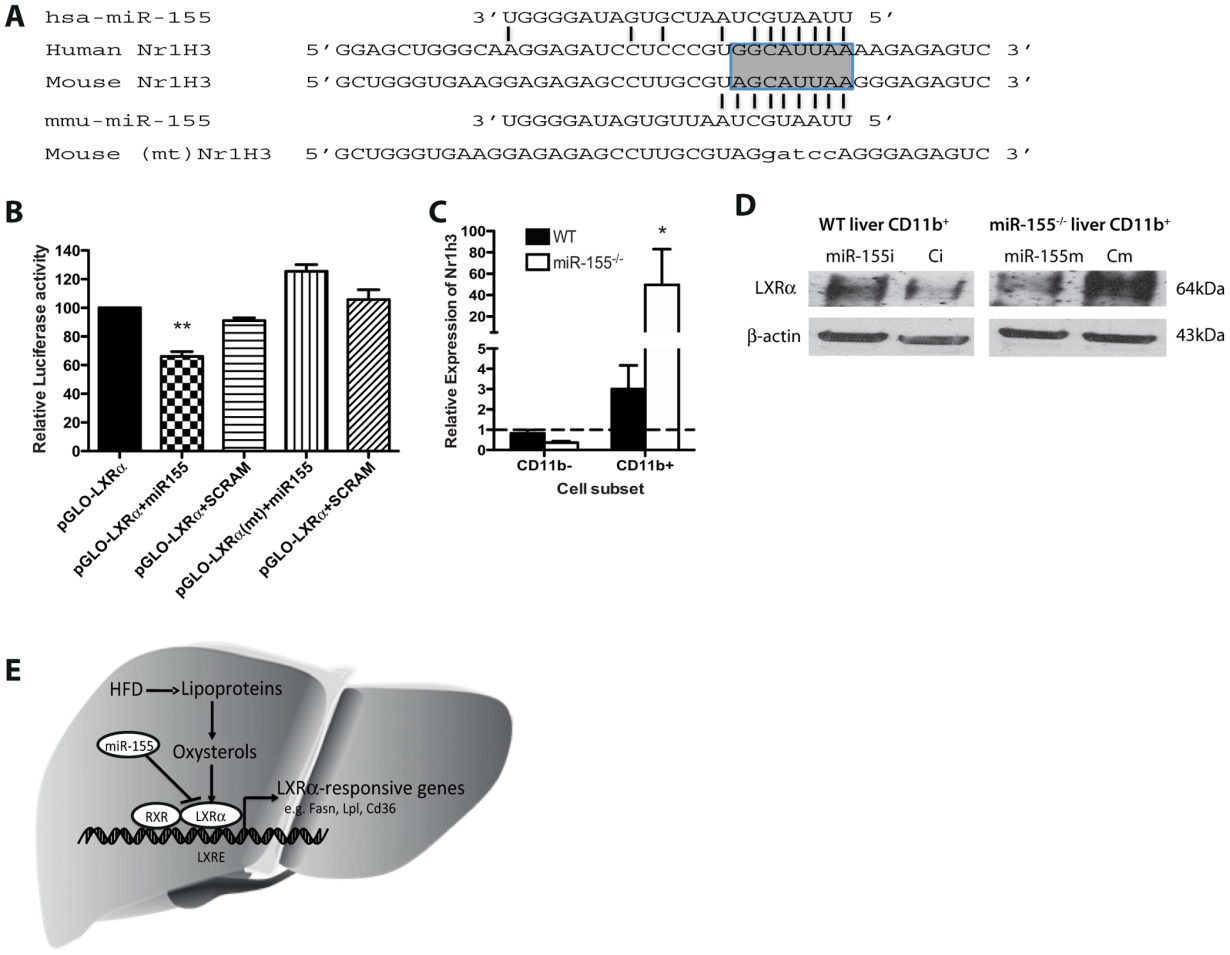
Identification of Nr1h3 (LXRα) as a miR-155 target gene. (**A**) Schematic showing alignment of mouse and human LXR 3′UTR with the miR155 seed region highlighted. (**B**) miR-155 binds directly to the 3′UTR of mouse *Nr1h3* (LXRα) mRNA. Murine pGLO-LXRα 3′UTR luciferase plasmids were co-transfected with miR-155 or scrambled (SCRAM) control mimic (40 nM) in HEK293 cells. Plasmids with a mutated 3′UTR are shown as pGLO-LXRα(mt). Luciferase activity was analyzed at 24 h. Data are Means ± SEM, n = 3 from 2 independent experiments. (**C**) Expression of *Nr1h3* (LXRα) in bead sorted CD11b^+^ macrophages and CD11b^−^ cells from livers of WT and miR-155^−/−^ mice (n = 3). * p<0.05, ** p<0.01 Student’s unpaired *t*-test. (**D**) Western blot for LXRα in CD11b^+^ cells isolated from WT or miR-155^−/−^ livers and transfected with control mimic (Cm), miR-155 mimic, control inhibitor (Ci) or miR-155 inhibitor. β-actin is shown as a loading control. (**E**) Schematic diagram indicating the pathway of miR-155 inhibition of LXRα-induced gene regulation of lipid accumulation during hepatic steatosis following a high fat diet (HFD).

Next, in order to identify the direct molecular mechanism responsible for miR-155 action, we combined bioinformatics and transcriptomic profiling of miR-155^−/−^ livers as follows. Firstly, we identified putative binding sites for miR-155 in the 3′UTR of 440 genes (TargetScanHuman v6.2) that were also conserved across species. By comparing this list with our transcriptomic data (Table S1) in Ingenuity Pathway Analysis, 7 genes emerged as potential direct miR-155 target genes that were implicated in liver metabolism (*Abcd2, Lpl, Pla2g7, Agtrap, Msr1, Nr1h3 and Ywhae*) for further validation (Figure 4A, Tables S3 & S4). We confirmed by qPCR that 4 of these potential targets were strongly upregulated in miR-155^−/−^ livers (Figure 4B). Next, we tested the direct interactions of miR-155 with 3′UTR of these mRNAs in luciferase reporter assays. Among the potential targets examined, *Nr1h3* (LXRα) was identified as a direct target of mouse miR-155 and the binding site appears to be conserved in humans (Figure 5A). The evidence for this is shown in Figure 5B - significant inhibition of luciferase activity was observed in HEK293 cells co-transfected with plasmids for the 3′UTR of mouse *Nr1h3* (LXRα) and the corresponding miR-155 mimic (pGLO-LXRa+miR155) versus scramble control mimic (pGLO-LXRa+SCRAM). Mutation of the *Nr1h3* (LXR α) miR-155 target sequence prevented downregulation of luciferase activity by miR-155 mimic (pGLO-LXRa(mt)+miR155) (Figure 5B), thus confirming *Nr1h3* (LXRα) as a *bonafide* miR-155 target gene. To identify the cell type where the miR-155/LXRα pathway is operating in the liver, we examined the expression of LXRα in CD11b^+^ and CD11b^−^ hepatic fractions. *Nr1h3* (LXRα) was upregulated only in CD11b^+^ macrophages, and not in CD11b^−^ cells, purified from livers of miR-155^−/−^ mice compared WT control (Figure 5C). These data suggest that the effect of miR-155 absence on *Nr1h3* (LXRα) upregulation may be taking place in Kupffer cells, and not in other liver cell lineages. To investigate this further we isolated CD11b^+^ cells from liver of WT and miR-155^−/−^ mice and transfected them with control or miR-155 mimics, control or miR-155 inhibitors (Figure 5D). Western blotting for LXRα demonstrated that transfection with a miR-155 inhibitor in CD11b^+^ cells from WT livers resulted in an upregulation of LXRα protein. In contrast, transfection with a miR-155 mimic in CD11b^+^ cells from miR-155^−/−^ livers resulted in downregulation of LXRα.

In addition, we found that the LXR-responsive genes *Fasn, Cd36* and *Lpl* (Figure 3C, Figure 4B) were upregulated in livers of miR-155^−/−^ mice but not all LXRα target genes were altered in the livers of miR-155^−/−^ mice, despite changes in LXRα expression. For example, *Cyp7a1* and *Srebf1* did not change (Figure 3C). However, a loss of repression of LXRα expression in miR-155^−/−^ mice does represent a plausible mechanism by which the hepatic phenotype is induced (Figure 5E).

Using luciferase reporter assays the other predicted miR-155 targets (*Abcd2*, *Lpl, Pla2g7, Agtrap, Msr1* or *Ywhae*) could not be validated as direct targets of miR-155 (Figure S2). Therefore, upregulation of these genes in livers of miR-155^−/−^ mice is likely to be a result of secondary effects of deregulation of the miR-155/LXRα pathway.

## Discussion

There is growing evidence for a role of miRNAs in the pathogenesis of many liver diseases [5], and recent studies have shown that expression of miR-155 is increased in hepatitis C virus infection [16], alcoholic liver disease [17], HCC [18], and in NAFLD [6], [7]. However, it has remained unclear whether miR-155 functionally contributes to development of liver disease. Here we report for the first time that absence of miR-155 in mice fed HFD for 6 months is associated with a significant increase in hepatic steatosis, suggesting a protective role of miR-155 in liver lipid metabolism.

Our data demonstrate that miR-155 was upregulated in livers of obese mice, and that this increased expression was primarily detected in CD11b^+^ macrophage cells. Inflammatory mediators (e.g. LPS, TNFα) have previously been shown to increase expression of miR-155 in macrophages and fibroblasts [19], [20], thus given the strong pro-inflammatory environment within the steatotic liver it is likely that inflammatory signals can upregulate miR-155. Furthermore, previous studies have shown that diet-induced activation of NF-κB is critical mediator regulating expression of miR-155 [6]. In addition, we would like to speculate that lipids themselves might directly regulate expression of miR-155. Analysis of the human and mouse miR-155 promoter (MATInspector) reveals multiple binding sites for LXR/RXR heterodimers indicating that miR-155 could be directly induced by oxysterols generated as part of a HFD. This may comprise a counterbalance mechanism, thus preventing excessive liver lipid overload and warrants further investigation.

Although miR-155 is generally considered a pro-inflammatory miRNA in macrophages during chronic inflammatory diseases such as rheumatoid arthritis [19], [20], [21], the data herein suggests that increased expression of miR-155 in obese liver macrophages is an important part of a protective negative regulatory feedback mechanism aimed at limiting disease progression by preventing an excessive lipid accumulation in the liver. In fact, in the context of lipid and inflammatory signaling in macrophages conflicting data has been obtained for miR-155. For example, miR-155 induced the chemokine CCL2 in macrophages stimulated with mildly oxLDL (moxLDL) and IFN-γ, but not highly oxidized LDL, via direct suppression of *Bcl6*, a transcription factor that counter-regulates NF-κB activation, thus indicating pro-inflammatory actions in the context of atherosclerotic plaque macrophages [22]. However, other studies show contrasting anti-inflammatory effects of miR-155 in lipid-laden macrophages. Silencing of endogenous miR-155 in macrophages significantly enhanced oxLDL-induced lipid uptake, up-regulated the expression of scavenger receptors (LOX-1, CD36 and CD68), and promoted the release of several cytokines including IL-6, -8, and TNF-α [23]. Chen *et al* demonstrated similar findings showing that inhibition of miR-155 expression significantly induced lipid uptake and over-expression miR-155 can decrease the lipid uptake in PMA-differentiated THP-1 cells and dendritic cells [24]. Conflicting data has also been obtained using *in vivo* murine atherosclerosis models where one study showed that haematopoietic deficiency of miR-155 in LDLR^−/−^ mice led to enhanced early atherosclerosis due to an increase in neutrophil migration [25], while another study more recently demonstrated that haematopoietic deficiency of miR-155 in ApoE^−/−^ mice led to decreased atherosclerosis via inhibition of *Bcl6* in macrophages [22]. Thus, it is clear that miR-155 can have different effects on macrophage lipid uptake and inflammatory signals in different settings. In fact, we observe a relative minor difference in inflammatory cytokines between WT and miR-155^−/−^ mice on HFD (a decrease in IL-1β, no change in IL-6 and TNFα) suggesting miR-155 preferentially regulates lipid metabolism pathways in these cells. This may reflect a role in fine-tuning of macrophage function that is context, activation status and tissue dependent on the availability of mRNA targets, and other miR binding and competing RNAs in the macrophage [26].

MiRNAs are critical regulators of many pathways by negatively targeting expression of multiple genes. Our studies suggest that the LXR and LXR-regulated genes are highly induced in livers, and liver macrophages, from miR-155^−/−^ mice, and using luciferase assays we show that LXRα is a direct molecular target of miR-155. LXRs are a class of nuclear receptors activated by endogenous oxysterols [9], and previous studies have shown that the LXRα gene is upregulated in liver of NAFLD and Hepatitis C patients who had steatosis [27]. Furthermore, several previous studies have shown that treatment of mice with pharmacological LXR agonists induces fatty liver [28]. This appears to be mediated via an induction of fatty acid synthesis and the scavenger receptor CD36, contributing to hepatic lipid accumulation [29]. Concordantly, both of these genes are also upregulated in miR-155^−/−^ livers in our study. Thus, we speculate that LXRα upregulation in the absence of repression by miR-155 leads to excessive lipid accumulation in liver. However, not all LXRα target genes were altered in miR-155^−/−^ livers, despite changes in LXRα expression (e.g. *Cyp7a1* and *Srebp1*). We cannot exclude the possibility that these genes may be altered at other time points in the model and thus contribute to the observed phenotype. Furthermore, miRNAs are thought to be involved in fine-tuning pathways rather than strong regulation, and do so most likely as part of a composite regulatory network. Thus, we speculate that the magnitude of depression of LXRα in miR-155^−/−^ is able to activate some but not all LXR dependent pathways.

Recent studies also suggest that miR-155 may have metabolic effects beyond liver. Expression of miR-155 in sub-cutaneous fat biopsies was significantly higher in samples from normal glucose control subjects as compared to those with T2D [30]. In cell-based experiments incubation of 3T3-L1 adipocytes with insulin resulted in a significant decrease in miR-155 [31]. Furthermore, expression of miR-155 is decreased during adipogenesis *in vitro* and over-expression of miR-155 inhibited expression of PPARγ and cEBPα, thus suggesting that miR-155 acts as a negative regulator of adipogenesis [32]. Finally, molecular studies showed that miR-155 could directly target proteins that were implicated in metabolism. For example, miR-155 inhibits translation of c-EBPβ (protein involved in early adipogenesis) and SOCS-1 (inhibitor of insulin signaling) in macrophages [15], [33], thus indicating a potential regulatory role of miR-155 in other metabolic pathways [23]. In fact, we observed a strong increase in *Socs1* expression in livers of miR-155^−/−^ mice suggesting the presence of functional interaction between miR-155 and *Socs1* in liver. Given the potent role of *Socs1* in inhibiting insulin signaling, it is likely that de-repression of *Socs1* in miR-155^−/−^ livers is responsible for an increase in the circulating insulin levels. Therefore, it was surprising that the insulin tolerance was not altered in the miR-155^−/−^ mice. However, this may be a reflection of the model of diet-induced obesity used, as it is not a model of type 2 diabetes. Or it may be a result of the time point the insulin tolerance test was carried out when other compensatory pathways may have been initiated. A more detailed analysis of glucose responses over several weeks following inhibition of miR-155 is warranted in future studies in mouse models of diabetes. In addition, strong up-regulation of *Nos2* expression in miR-155^−/−^ liver was detected. In contrast to endothelial NOS (*Nos3*) [34], *Nos2* is not predicted to be targeted by miR-155 (TargetScan), and LXR has previously been implicated in transrepression of *Nos2* rather than its activation [35]. Thus, altered *Nos2* expression is likely to be simply a consequence of fatty liver pathology in these mice.

In summary, we have shown that miR-155 has a novel regulatory role as an important regulator of cholesterol and lipid metabolism, operating at least in liver via the LXRα pathway. Increased expression of miR-155 in models of NAFLD likely plays a critical homeostatic role designed to prevent excessive lipid accumulation in livers that can ultimately lead to liver damage. Future studies determining how miR-155 may interact with lipid, fibrosis and inflammatory pathways in liver (and other metabolic tissues) are warranted and could offer new insights into the pathogenesis of hepatic steatosis and type 2 diabetes.

## Supporting Information

Figure S1 **Absence of miR-155 does not affect fasting glucose levels or responses in an Insulin Tolerance Test.** (**A**) Fasting glucose (mmol/L) and (**B**) insulin tolerance tests at week 24 in WT or miR-155^−/−^ mice fed either chow or HFD. Data are Means ± SEM pooled from 2 independent experiments, n = 13–21 mice/group.
(EPS)

Figure S2 **miR-155 does not target the 3′UTR of Abcd2, Lpl, Pla2g7, Agtrap, Msr1 or Ywhae mRNAs.** Mouse pGLO-(gene of interest) 3′UTR luciferase plasmids were co-transfected with miR-155 or scrambled (SCRAM) control mimic (40 nM) in HEK293 cells. Luciferase activity was analyzed at 24 h. Data are Means ± SEM, n = 3 from 2 independent experiments.
(EPS)

Table S1 **List of differentially expressed genes obtained by microarray analysis of livers from WT vs miR-155^−/−^ mice.** Genes further validated by qRT-PCR are marked in bold type.
(DOCX)

Table S2 **The top five significantly upregulated canonical pathways in livers from miR-155^−/−^ mice compared to WT livers as assessed by use of Ingenuity Pathway Analysis.**
(DOCX)

Table S3 **List of potential miR-155 targets chosen for further validation.** The miR-155 predicted targets in mouse and/or human were identified according to various target prediction algorithms and were further chosen for validation because they were also identified by microarray analysis or had known links to liver/lipid/fibrosis pathways as indicated by Ingenuity pathway analysis (IPA).
(DOCX)

Table S4 **List of primer sequences designed for the potential murine miR-155 targets chosen for further validation.**
(DOCX)
