# Supplementary material for: MiR-155 Has a Protective Role in the Development of Non-Alcoholic Hepatosteatosis in Mice
Source: PLoS One. 2013 Aug 21;8(8):e72324. doi: 10.1371/journal.pone.0072324 (PMC3749101; doi:10.1371/journal.pone.0072324)
Supplement: Table S1 — List of differentially expressed genes obtained by microarray analysis of livers from WT vs miR-155−/− mice. Genes further validated by qRT-PCR are marked in bold type. (DOCX) [file pone.0072324.s003.docx]

| **Gene** | **Probeset ID** |  | **Log Ratio** | **p-value** |
| --- | --- | --- | --- | --- |
| *Gpnmb* | 10538187 |  | 4.938 | 0.00E+00 |
| *Ly6d* | 10429520 |  | 4.582 | 0.00E+00 |
| *Lcn2* | 10481627 |  | 3.972 | 0.00E+00 |
| *Mt2a* | 10574023 |  | 3.462 | 0.00E+00 |
| *Mmp12* | 10583056 |  | 3.397 | 0.00E+00 |
| *Dpt* | 10351293 |  | 3.186 | 4.00E-04 |
| *Atp6v0d2* | 10511779 |  | 3.121 | 1.20E-03 |
| *Emp1* | 10542355 |  | 3.12 | 1.20E-03 |
| *Cyp4a14* | 10515187 |  | 3.115 | 1.10E-03 |
| *Clec7a* | 10548375 |  | 3.101 | 1.30E-03 |
| *Gpx3* | 10376201 |  | 3.062 | 1.10E-03 |
| *Timp1* | 10598976 |  | 3.01 | 1.00E-03 |
| *Orm1/Orm2* | 10505451 |  | 2.988 | 1.00E-03 |
| *Mybl1* | 10353010 |  | 2.976 | 1.00E-03 |
| *SPP1 (Includes EG:20750)* | 10523717 |  | 2.967 | 1.10E-03 |
| *Lgals3* | 10414360 |  | 2.933 | 1.20E-03 |
| *Acot2* | 10397145 |  | 2.929 | 1.30E-03 |
| *Saa1* | 10563597 |  | 2.929 | 1.10E-03 |
| *Cd24a* | 10362896 |  | 2.915 | 1.10E-03 |
| *D17h6s56e-5* | 10450374 |  | 2.828 | 1.50E-03 |
| *Aqp7* | 10512145 |  | 2.787 | 1.90E-03 |
| *Elovl7* | 10407072 |  | 2.772 | 1.90E-03 |
| *S100a11* | 10457640 |  | 2.763 | 1.90E-03 |
| *Cdkn1a* | 10443463 |  | 2.762 | 2.10E-03 |
| *Lum* | 10365983 |  | 2.747 | 1.80E-03 |
| *Plp2* | 10473022 |  | 2.73 | 2.00E-03 |
| ***Abcd2*** | 10431697 |  | 2.708 | 1.90E-03 |
| *Cd63* | 10367436 |  | 2.694 | 1.90E-03 |
| *Prrg4* | 10485624 |  | 2.619 | 2.50E-03 |
| *Mt1e* | 10574027 |  | 2.608 | 2.20E-03 |
| *Olfml3* | 10500808 |  | 2.605 | 2.30E-03 |
| *Cyp21a2* | 10450272 |  | 2.604 | 2.30E-03 |
| *Glipr1* | 10372410 |  | 2.516 | 2.70E-03 |
| *Gstm3* | 10501218 |  | 2.505 | 3.70E-03 |
| *Ccnd1* | 10569646 |  | 2.469 | 3.40E-03 |
| *Cxcl10* | 10531415 |  | 2.465 | 3.40E-03 |
| *Cidec* | 10546929 |  | 2.454 | 5.20E-03 |
| *Ccl21* | 10512377 |  | 2.42 | 4.20E-03 |
| *Asns* | 10543067 |  | 2.408 | 3.60E-03 |
| *Saa2* | 10553274 |  | 2.384 | 4.60E-03 |
| *Defb1* | 10570741 |  | 2.328 | 5.60E-03 |
| *Fstl1* | 10435641 |  | 2.319 | 4.80E-03 |
| *Ms4a6d* | 10466210 |  | 2.313 | 5.10E-03 |
| *Ms4a7* | 10466200 |  | 2.273 | 5.50E-03 |
| *Tinag* | 10595081 |  | 2.268 | 5.40E-03 |
| *Anxa13* | 10428814 |  | 2.259 | 5.80E-03 |
| *Cd68* | 10387536 |  | 2.25 | 5.80E-03 |
| *Usp18* | 10541307 |  | 2.249 | 5.60E-03 |
| *Ccnb2* | 10594774 |  | 2.248 | 5.70E-03 |
| *Ccl3l1/Ccl3l3* | 10389231 |  | 2.247 | 5.80E-03 |
| *Sh3bgrl3* | 10517169 |  | 2.236 | 6.10E-03 |
| *Ear2 (Includes Others)* | 10414262 |  | 2.231 | 5.70E-03 |
| *Gm5068* | 10396440 |  | 2.23 | 6.20E-03 |
| *Cd34* | 10352905 |  | 2.209 | 5.80E-03 |
| *Hla-Dqa1* | 10450154 |  | 2.206 | 7.50E-03 |
| *Ccl6* | 10389222 |  | 2.2 | 6.40E-03 |
| *Mfge8* | 10564713 |  | 2.188 | 6.10E-03 |
| *Dbp* | 10553092 |  | 2.186 | 7.80E-03 |
| *Fabp7* | 10363224 |  | 2.172 | 7.30E-03 |
| *Sprr1a* | 10499899 |  | 2.167 | 7.30E-03 |
| *Plac8* | 10531724 |  | 2.165 | 7.40E-03 |
| *Apoa4* | 10593174 |  | 2.146 | 6.60E-03 |
| *Fcer1g* | 10360070 |  | 2.11 | 8.40E-03 |
| *Retnlb* | 10436100 |  | 2.097 | 8.30E-03 |
| *Prss8* | 10568332 |  | 2.088 | 8.40E-03 |
| *Cd74* | 10456005 |  | 2.072 | 1.13E-02 |
| *Rgs10* | 10568392 |  | 2.07 | 9.00E-03 |
| *Ly6a (Includes Others)* | 10429568 |  | 2.064 | 1.02E-02 |
| *Cd72* | 10512470 |  | 2.062 | 1.02E-02 |
| *Wee1* | 10556266 |  | 2.004 | 1.14E-02 |
| *Fxyd5* | 10562192 |  | 1.999 | 1.21E-02 |
| *Pqlc3* | 10399540 |  | 1.996 | 1.14E-02 |
| *RGS2 (Includes EG:19735)* | 10358389 |  | 1.996 | 1.13E-02 |
| *Ms4a6a* | 10461622 |  | 1.993 | 1.24E-02 |
| *Usp2* | 10584634 |  | 1.984 | 1.15E-02 |
| *1600029D21Rik* | 10585186 |  | 1.978 | 1.15E-02 |
| *Emp2* | 10437639 |  | 1.975 | 1.14E-02 |
| *Ifi27l2* | 10402347 |  | 1.969 | 1.24E-02 |
| *Mad2l1* | 10538832 |  | 1.96 | 1.29E-02 |
| *Fam83b* | 10595070 |  | 1.943 | 1.29E-02 |
| *C14orf105* | 10419392 |  | 1.934 | 1.31E-02 |
| *Ifi30* | 10579347 |  | 1.932 | 1.47E-02 |
| *Tmem86a* | 10553324 |  | 1.926 | 1.28E-02 |
| *H19* | 10569335 |  | 1.92 | 1.31E-02 |
| *Cdk1* | 10369815 |  | 1.914 | 1.31E-02 |
| *Kdelr3* | 10425287 |  | 1.91 | 1.41E-02 |
| *Emp3* | 10563441 |  | 1.909 | 1.66E-02 |
| *Vim* | 10469322 |  | 1.901 | 1.42E-02 |
| *Scara3* | 10420891 |  | 1.9 | 1.39E-02 |
| *Anxa3* | 10523451 |  | 1.893 | 1.44E-02 |
| *Gsta5* | 10587331 |  | 1.89 | 1.38E-02 |
| *Casp12* | 10583008 |  | 1.888 | 1.48E-02 |
| *Ccna2* | 10497831 |  | 1.888 | 1.42E-02 |
| *Itgax* | 10557895 |  | 1.883 | 1.47E-02 |
| *Muc1* | 10493474 |  | 1.881 | 1.46E-02 |
| *Tyrobp* | 10551883 |  | 1.877 | 1.62E-02 |
| *C1orf51* | 10373452 |  | 1.872 | 1.66E-02 |
| *Ccdc80* | 10435948 |  | 1.871 | 1.44E-02 |
| *Nt5dc2* | 10413710 |  | 1.868 | 1.60E-02 |
| *Raet1b* | 10362091 |  | 1.867 | 2.14E-02 |
| *Capn6* | 10607143 |  | 1.864 | 1.62E-02 |
| *Tagln2* | 10351825 |  | 1.86 | 1.61E-02 |
| *Hla-Dma* | 10444229 |  | 1.859 | 1.79E-02 |
| *Cd209* | 10576784 |  | 1.848 | 1.75E-02 |
| *Cd9* | 10548030 |  | 1.846 | 1.63E-02 |
| *Bcl2a1* | 10587683 |  | 1.842 | 1.70E-02 |
| *Anxa2* | 10586744 |  | 1.833 | 1.64E-02 |
| *Slc16a5* | 10382532 |  | 1.832 | 1.75E-02 |
| *LSP1 (Includes EG:16985)* | 10559207 |  | 1.829 | 1.74E-02 |
| *Tcf19* | 10450519 |  | 1.826 | 1.70E-02 |
| *Cygb* | 10393364 |  | 1.822 | 1.73E-02 |
| *Ly86* | 10404606 |  | 1.821 | 1.79E-02 |
| *Cyba* | 10582303 |  | 1.82 | 1.74E-02 |
| *Cbr3* | 10436978 |  | 1.817 | 1.65E-02 |
| *Actg2* | 10467124 |  | 1.811 | 1.75E-02 |
| *Sgce* | 10542965 |  | 1.808 | 1.74E-02 |
| *Ccnb1/Gm5593* | 10515836 |  | 1.805 | 1.69E-02 |
| *Csrp1* | 10350136 |  | 1.8 | 1.76E-02 |
| *Cd52* | 10517165 |  | 1.799 | 1.90E-02 |
| *Cyp2a12/Cyp2a22* | 10561162 |  | 1.796 | 1.90E-02 |
| *Rras* | 10552824 |  | 1.792 | 1.79E-02 |
| *Ctss* | 10494271 |  | 1.788 | 1.74E-02 |
| *Acot1* | 10397148 |  | 1.785 | 1.73E-02 |
| *Hla-Dqb1* | 10444291 |  | 1.785 | 1.89E-02 |
| *Abhd2* | 10554269 |  | 1.78 | 1.78E-02 |
| *Trim30a/Trim30d* | 10566366 |  | 1.779 | 1.79E-02 |
| *Crip1* | 10399005 |  | 1.775 | 1.91E-02 |
| *Laptm5* | 10508663 |  | 1.775 | 1.90E-02 |
| *Mmp2* | 10573924 |  | 1.774 | 1.74E-02 |
| *Gpr137b* | 10407792 |  | 1.773 | 1.84E-02 |
| *Atf3* | 10361091 |  | 1.77 | 1.89E-02 |
| *Gm885* | 10382106 |  | 1.767 | 1.90E-02 |
| *Trem2* | 10445781 |  | 1.764 | 1.89E-02 |
| *Kcne3* | 10555297 |  | 1.76 | 1.91E-02 |
| *Rhoc* | 10495054 |  | 1.757 | 1.90E-02 |
| *Ifi44* | 10502791 |  | 1.756 | 2.00E-02 |
| *Tlr13* | 10601385 |  | 1.754 | 1.91E-02 |
| ***Pla2g7*** | 10445293 |  | 1.745 | 1.90E-02 |
| *Ccr2* | 10590631 |  | 1.744 | 1.95E-02 |
| *Golm1* | 10409767 |  | 1.742 | 1.85E-02 |
| *C3ar1* | 10547657 |  | 1.736 | 1.89E-02 |
| *Arpc1b* | 10527441 |  | 1.735 | 1.99E-02 |
| *Tmem45a* | 10440019 |  | 1.733 | 1.90E-02 |
| *Hist1h2ad* | 10408077 |  | 1.721 | 1.94E-02 |
| *Slc7a11* | 10498024 |  | 1.719 | 1.95E-02 |
| *Fabp5* | 10585699 |  | 1.717 | 2.16E-02 |
| *Ect2* | 10497520 |  | 1.716 | 1.96E-02 |
| *Mcm6* | 10357436 |  | 1.716 | 1.91E-02 |
| *Sirpb1* | 10497358 |  | 1.715 | 2.00E-02 |
| *Sparcl1* | 10531931 |  | 1.715 | 1.96E-02 |
| *Nrg4* | 10593776 |  | 1.714 | 2.07E-02 |
| *Cln6* | 10586110 |  | 1.708 | 2.15E-02 |
| *Capg* | 10539135 |  | 1.702 | 2.19E-02 |
| *Ifit3* | 10462618 |  | 1.7 | 2.14E-02 |
| *Hla-Drb1* | 10444298 |  | 1.695 | 2.39E-02 |
| *Fcgr2a* | 10360040 |  | 1.693 | 2.16E-02 |
| *Serpinb6* | 10408600 |  | 1.692 | 2.06E-02 |
| *Rfc4* | 10438690 |  | 1.688 | 2.23E-02 |
| *Gm609* | 10439660 |  | 1.685 | 2.31E-02 |
| *Tspan8* | 10366446 |  | 1.673 | 2.24E-02 |
| *Dtl* | 10361110 |  | 1.67 | 2.14E-02 |
| *C11orf86* | 10464772 |  | 1.669 | 2.47E-02 |
| *Tmsb10/Tmsb4x* | 10551009 |  | 1.663 | 2.38E-02 |
| *Epcam* | 10447383 |  | 1.662 | 2.24E-02 |
| *Ccl5* | 10389207 |  | 1.657 | 2.47E-02 |
| *Mustn1* | 10413609 |  | 1.656 | 2.48E-02 |
| *Acot1* | 10397158 |  | 1.653 | 2.47E-02 |
| *Tmem43* | 10540105 |  | 1.648 | 2.34E-02 |
| *Ucp2* | 10555389 |  | 1.648 | 2.53E-02 |
| *Il1rn* | 10469816 |  | 1.647 | 2.40E-02 |
| *Npdc1* | 10470027 |  | 1.647 | 2.47E-02 |
| *Zdhhc2* | 10571399 |  | 1.647 | 2.35E-02 |
| *Aldh1b1* | 10504606 |  | 1.644 | 2.38E-02 |
| *Ms4a6c* | 10461614 |  | 1.64 | 2.47E-02 |
| *Cstb* | 10364375 |  | 1.639 | 2.47E-02 |
| *Blnk* | 10467508 |  | 1.636 | 2.45E-02 |
| *Cbr1* | 10436967 |  | 1.636 | 2.46E-02 |
| *Cdkn2c* | 10515090 |  | 1.635 | 2.39E-02 |
| *Oasl2* | 10524621 |  | 1.632 | 2.47E-02 |
| *Fam83a* | 10424245 |  | 1.631 | 2.50E-02 |
| *Il2rg* | 10606016 |  | 1.631 | 2.83E-02 |
| *Chaf1b* | 10437040 |  | 1.63 | 2.53E-02 |
| *Gas6* | 10577164 |  | 1.629 | 2.41E-02 |
| *Mtmr11* | 10494351 |  | 1.626 | 2.40E-02 |
| *Abcb1* | 10519527 |  | 1.621 | 2.46E-02 |
| *Alox5ap* | 10527638 |  | 1.619 | 2.51E-02 |
| *Slc25a4* | 10578539 |  | 1.618 | 2.67E-02 |
| *Osbpl3* | 10544660 |  | 1.615 | 2.73E-02 |
| *Ifi204 (Includes Others)* | 10360377 |  | 1.614 | 2.75E-02 |
| *Il17rb* | 10418341 |  | 1.614 | 2.50E-02 |
| *Gpx8* | 10412207 |  | 1.613 | 2.52E-02 |
| *Renbp* | 10605181 |  | 1.608 | 2.73E-02 |
| *C15orf23* | 10474825 |  | 1.598 | 2.51E-02 |
| *Igsf6* | 10567580 |  | 1.598 | 2.90E-02 |
| *Stmn1* | 10513818 |  | 1.596 | 2.53E-02 |
| *Trim12a* | 10566326 |  | 1.596 | 2.64E-02 |
| *Cdc42ep5* | 10559509 |  | 1.593 | 2.93E-02 |
| *Hexb* | 10411373 |  | 1.593 | 2.85E-02 |
| *Hist1h2ab/Hist1h2ae* | 10404063 |  | 1.576 | 2.84E-02 |
| *Rbp1* | 10588037 |  | 1.574 | 2.61E-02 |
| ***Lpl*** | 10572130 |  | 1.573 | 2.91E-02 |
| *Lyz1/Lyz2* | 10372648 |  | 1.571 | 3.13E-02 |
| *C1qc* | 10517513 |  | 1.567 | 3.27E-02 |
| *Pole2* | 10400649 |  | 1.563 | 2.86E-02 |
| *Rcn3* | 10563077 |  | 1.563 | 3.24E-02 |
| *Klf6* | 10403352 |  | 1.557 | 2.92E-02 |
| *Pygb* | 10476969 |  | 1.553 | 2.81E-02 |
| *Frzb* | 10484307 |  | 1.548 | 3.20E-02 |
| *C19orf38* | 10583669 |  | 1.543 | 3.30E-02 |
| *Gadd45b* | 10364950 |  | 1.543 | 2.86E-02 |
| *Rab18* | 10453715 |  | 1.543 | 3.39E-02 |
| *Hspb1* | 10526410 |  | 1.53 | 3.55E-02 |
| *Id1* | 10477169 |  | 1.528 | 3.24E-02 |
| *Plek* | 10384458 |  | 1.524 | 3.39E-02 |
| *Ms4a4b (Includes Others)* | 10461594 |  | 1.523 | 3.57E-02 |
| *Vcam1* | 10501608 |  | 1.517 | 3.40E-02 |
| *Chek1* | 10592201 |  | 1.516 | 3.31E-02 |
| *Gldn* | 10585398 |  | 1.514 | 3.28E-02 |
| *Cd53* | 10501063 |  | 1.513 | 3.56E-02 |
| *Fam180a* | 10543939 |  | 1.51 | 3.46E-02 |
| *Tm4sf4* | 10492174 |  | 1.506 | 3.33E-02 |
| *Alpl* | 10517587 |  | 1.504 | 3.41E-02 |
| *Scd2* | 10463355 |  | 1.504 | 3.43E-02 |
| *Tmem173* | 10458314 |  | 1.503 | 3.53E-02 |
| *Nid1* | 10403584 |  | 1.498 | 3.43E-02 |
| *Anxa5* | 10497817 |  | 1.493 | 3.81E-02 |
| *Bicc1* | 10369844 |  | 1.493 | 3.48E-02 |
| *Lpcat2* | 10573939 |  | 1.493 | 3.52E-02 |
| *Gstt2/Gstt2b* | 10370013 |  | 1.49 | 3.57E-02 |
| *Lox* | 10458894 |  | 1.488 | 3.44E-02 |
| *Pmepa1* | 10490159 |  | 1.485 | 3.68E-02 |
| *TIMP2 (Includes EG:21858)* | 10393559 |  | 1.485 | 3.56E-02 |
| *Plat* | 10570855 |  | 1.482 | 3.47E-02 |
| *Cyp4f16/Gm9705* | 10443869 |  | 1.477 | 3.57E-02 |
| *Bcl2a1c* | 10589884 |  | 1.476 | 3.90E-02 |
| *Tmem176a* | 10538150 |  | 1.474 | 3.81E-02 |
| *Sparc* | 10386058 |  | 1.472 | 3.73E-02 |
| *Oas1* | 10533246 |  | 1.471 | 3.74E-02 |
| *Slamf7* | 10360173 |  | 1.471 | 3.89E-02 |
| *Scn2a* | 10472400 |  | 1.465 | 3.82E-02 |
| *Slc25a24* | 10495405 |  | 1.46 | 3.79E-02 |
| *Clec12a* | 10542164 |  | 1.457 | 4.20E-02 |
| *Ccdc3* | 10469066 |  | 1.451 | 3.90E-02 |
| *Ube2c* | 10478572 |  | 1.449 | 4.02E-02 |
| *Ppap2c* | 10370552 |  | 1.447 | 4.20E-02 |
| *Slc39a4* | 10430006 |  | 1.447 | 4.07E-02 |
| *Uap1l1* | 10480714 |  | 1.445 | 4.03E-02 |
| *Cmtm3* | 10574471 |  | 1.444 | 4.41E-02 |
| *HIST1H3A (Includes Others)* | 10408239 |  | 1.443 | 4.02E-02 |
| *Cenpn* | 10575733 |  | 1.436 | 4.21E-02 |
| *Slpi* | 10489463 |  | 1.433 | 4.13E-02 |
| *Ifit1b* | 10462623 |  | 1.427 | 4.22E-02 |
| *Bmp8b* | 10507817 |  | 1.426 | 4.33E-02 |
| *Prelid2* | 10458589 |  | 1.425 | 4.22E-02 |
| *Hist3h3* | 10404049 |  | 1.42 | 4.32E-02 |
| *Itga8* | 10480090 |  | 1.418 | 4.34E-02 |
| *Rbm3* | 10556113 |  | 1.418 | 4.32E-02 |
| *Pigp* | 10545528 |  | 1.414 | 4.82E-02 |
| *Tuba8* | 10541301 |  | 1.413 | 4.72E-02 |
| *Cd48* | 10351658 |  | 1.412 | 4.63E-02 |
| *Dynlt1* | 10548785 |  | 1.411 | 4.65E-02 |
| *App* | 10440491 |  | 1.41 | 4.33E-02 |
| *Phlda3* | 10350146 |  | 1.406 | 4.44E-02 |
| *Ptafr* | 10508734 |  | 1.405 | 4.73E-02 |
| *TPM1 (Includes EG:22003)* | 10594661 |  | 1.404 | 4.58E-02 |
| *Emb* | 10407327 |  | 1.403 | 4.66E-02 |
| *Asf1b* | 10573261 |  | 1.395 | 4.59E-02 |
| *C11orf82* | 10565570 |  | 1.395 | 4.73E-02 |
| *Clec4a3* | 10541564 |  | 1.394 | 4.90E-02 |
| *Arhgdib* | 10548892 |  | 1.392 | 4.96E-02 |
| *Dynll1* | 10532984 |  | 1.39 | 4.73E-02 |
| *Gm3579* | 10573865 |  | 1.39 | 4.75E-02 |
| *Gdpd1* | 10389590 |  | 1.389 | 4.73E-02 |
| *C1qb* | 10517508 |  | 1.387 | 4.95E-02 |
| *Haus8* | 10579468 |  | 1.387 | 4.73E-02 |
| *Islr* | 10594044 |  | 1.387 | 4.83E-02 |
| *Aldh3b1* | 10464560 |  | 1.384 | 4.98E-02 |
| *Plk1* | 10557156 |  | 1.384 | 4.78E-02 |
| *Mastl* | 10480432 |  | 1.381 | 4.82E-02 |
| *Myof* | 10467258 |  | 1.38 | 4.68E-02 |
| *S100a6* | 10493820 |  | 1.379 | 4.96E-02 |
| *Fgf21* | 10563344 |  | 1.375 | 4.98E-02 |
| *Ca13* | 10490903 |  | 1.373 | 4.97E-02 |
| *Il10rb* | 10436841 |  | 1.371 | 4.98E-02 |
| *Cd180* | 10406928 |  | 1.366 | 4.91E-02 |
| *Gyg1* | 10497441 |  | 1.366 | 4.99E-02 |
| *Colec10* | 10424105 |  | -1.405 | 4.82E-02 |
| *Xlr3c (Includes Others)* | 10600122 |  | -1.429 | 4.49E-02 |
| *Hyou1* | 10584712 |  | -1.43 | 4.93E-02 |
| *Pdilt* | 10567394 |  | -1.436 | 4.90E-02 |
| *Ces2a* | 10574498 |  | -1.458 | 4.45E-02 |
| *Camk1d* | 10479852 |  | -1.461 | 4.89E-02 |
| *Slc15a5* | 10548931 |  | -1.47 | 4.18E-02 |
| *Gpr52* | 10359375 |  | -1.476 | 4.97E-02 |
| *P2ry4* | 10605938 |  | -1.517 | 3.72E-02 |
| *Gm10387* | 10379344 |  | -1.526 | 4.89E-02 |
| *Scnn1a* | 10541885 |  | -1.536 | 3.64E-02 |
| *Cyp2u1* | 10502214 |  | -1.54 | 3.63E-02 |
| *Slc25a25* | 10481634 |  | -1.545 | 4.44E-02 |
| *Chic1* | 10601312 |  | -1.553 | 3.43E-02 |
| *Slc30a10* | 10352548 |  | -1.586 | 3.06E-02 |
| *Slc17a8* | 10371796 |  | -1.595 | 3.00E-02 |
| *Kiaa1109* | 10491599 |  | -1.606 | 4.50E-02 |
| *Slc22a9* | 10465764 |  | -1.622 | 3.07E-02 |
| *Ido2* | 10577645 |  | -1.627 | 3.01E-02 |
| *Srebf1* | 10386473 |  | -1.659 | 2.65E-02 |
| *Tlcd2* | 10378572 |  | -1.702 | 2.13E-02 |
| *Nudt7* | 10575685 |  | -1.708 | 2.12E-02 |
| *Sdr9c7* | 10367066 |  | -1.733 | 1.98E-02 |
| *Cyp4f12* | 10449940 |  | -1.767 | 1.77E-02 |
| *Lifr* | 10422822 |  | -1.774 | 1.78E-02 |
| *Serpina12* | 10402428 |  | -1.799 | 1.53E-02 |
| *Gna14* | 10461856 |  | -1.834 | 1.50E-02 |
| *Ppp1r3c* | 10467206 |  | -1.903 | 1.47E-02 |
| *Fam55b* | 10593198 |  | -1.918 | 1.21E-02 |
| *Sucnr1* | 10492306 |  | -1.946 | 1.20E-02 |
| *Lrtm1* | 10413492 |  | -2.02 | 8.00E-03 |
| *Mup1 (Includes Others)* | 10513538 |  | -2.107 | 5.20E-03 |
| *Cyp7b1* | 10497381 |  | -2.113 | 5.20E-03 |
| *Fasn* | 10393970 |  | -2.118 | 5.00E-03 |
| *Ces3* | 10574607 |  | -2.132 | 5.20E-03 |
| *Trhde* | 10372421 |  | -2.281 | 4.20E-03 |
| *Rnase2* | 10419568 |  | -2.367 | 3.40E-03 |
| *Mir-505* | 10604832 |  | -2.405 | 1.20E-03 |
| *Thrsp* | 10565609 |  | -2.417 | 1.60E-03 |
| *Slc22a7* | 10451291 |  | -2.564 | 9.00E-04 |
| *Adh6-Ps1* | 10496462 |  | -2.586 | 7.00E-04 |
| *Obp2b* | 10470175 |  | -2.619 | 7.00E-04 |
| *Elovl3* | 10463551 |  | -2.912 | 2.00E-04 |
| *Susd4* | 10352439 |  | -3.033 | 0.00E+00 |
| *Cxcl13* | 10523359 |  | -3.124 | 0.00E+00 |
| *Cyp2c19* | 10467400 |  | -3.226 | 0.00E+00 |
| *2810007J24Rik* | 10560131 |  | -3.425 | 0.00E+00 |
| *Serpina4-Ps1* | 10398011 |  | -3.718 | 0.00E+00 |
| *Slco1a1* | 10548978 |  | -5.345 | 0.00E+00 |
| *Hsd3b4 (Includes Others)* | 10500545 |  | -6.764 | 0.00E+00 |
