# Supplementary material for: MiR-155 Has a Protective Role in the Development of Non-Alcoholic Hepatosteatosis in Mice
Source: PLoS One. 2013 Aug 21;8(8):e72324. doi: 10.1371/journal.pone.0072324 (PMC3749101; doi:10.1371/journal.pone.0072324)
Supplement: Table S2 — The top five significantly upregulated canonical pathways in livers from miR-155−/− mice compared to WT livers as assessed by use of Ingenuity Pathway Analysis. (DOCX) [file pone.0072324.s004.docx]

| **Canonical pathways up-regulated in miR-155^-/-^ livers** | **p value** |
| --- | --- |
| LXR/RXR activation  FXR/RXR activation  LPS/IL-1 mediated inhibition of RXR function  Hepatic cholestasis  Arachidonic acid metabolism | 2.97 x 10^-45^  1.69 x 10^-43^  3.04 x 10^-40^  1.4 x 10^-37^  3.56 x 10^-37^ |
