# Supplementary material for: MiR-155 Has a Protective Role in the Development of Non-Alcoholic Hepatosteatosis in Mice
Source: PLoS One. 2013 Aug 21;8(8):e72324. doi: 10.1371/journal.pone.0072324 (PMC3749101; doi:10.1371/journal.pone.0072324)
Supplement: Table S3 — List of potential miR-155 targets chosen for further validation. The miR-155 predicted targets in mouse and/or human were identified according to various target prediction algorithms and were further chosen for validation because they were also identified by microarray analysis or had known links to liver/lipid/fibrosis pathways as indicated by Ingenuity pathway analysis (IPA). (DOCX) [file pone.0072324.s005.docx]

| **Target gene** | **Full name** | **Software/**  **pathway** | **Prediction algorithm** |
| --- | --- | --- | --- |
| *Abcd2* | ATP-binding cassette, sub-family D | IPA/lipid | IPA miRTarBase |
| *Lpl* | Lipoprotein lipase | IPA/lipid | IPA miRTarBase |
| *Pla2g7* | Lipoprotein-associated phospholipase A2 group VII | IPA/lipid | PICTAR |
| *Agtrap* | Angiotensin II receptor associated protein | IPA/fibrosis | TargetScan |
| *Msr1* | Macrophage scavenger receptor 1 | IPA/liver | TargetScan |
| *Nr1h3* | Liver X receptor α | IPA/liver | TargetScan |
| *Ywhae* | 14-3-3 protein epsilon | IPA/liver | TargetScan |
