# Supplementary material for: MiR-155 Has a Protective Role in the Development of Non-Alcoholic Hepatosteatosis in Mice
Source: PLoS One. 2013 Aug 21;8(8):e72324. doi: 10.1371/journal.pone.0072324 (PMC3749101; doi:10.1371/journal.pone.0072324)
Supplement: Table S4 — List of primer sequences designed for the potential murine miR-155 targets chosen for further validation. (DOCX) [file pone.0072324.s006.docx]

| **Gene** | **PCR primers** |
| --- | --- |
| *Abcd2* | Fw agtttaaacTACAATTCGATGGCGAAGGAGGCT;  Rev agtcgacTTCTGCACAGCTGACATCAGTCCT |
| *Lpl* | Fw agtttaaacAACCCAGGGTGAGGAATCTAATGG  Rev agtcgacCTAGTTGCAAAGGCCAGGTGTTTC |
| *Pla2g7* | Fw agtttaaacCTCACCCAGGTCCCGGCTCAG  Rev agtcgacTGGCATCGTTCCTGACTAGAGGCT |
| *Agtrap* | Fw agtttaaacAACACACTCCTACTGAACTGCCCA;  Rev agtcgacTTCACAGATGCTGTCACTCAGCCT |
| *Msr1* | Fw agtttaaacCACCATGTTGTTTCCATGTTGATGCC;  Rev agtcgacCACAACAACCCTGTGAGGTAGGAA |
| *Nr1h3* | Fw agtttaaacTTCAGAGCAAGTGTTTGCCCTTCG;  Rev agtcgacACAAAGTGGCTCTCCTGGCTAGTT |
| *Ywhae* | Fw agtttaaacCCCATTGTCACTGAGAACCACCAA;  Rev agtcgacTGAGCAGCCACGGATTGTTGAACT |
